# Data sharing: A Long COVID perspective, challenges, and road map for the future

**DOI:** 10.17159/sajs.2023/14719

**Published:** 2023-05-30

**Authors:** Sunday O. Oladejo, Liam R. Watson, Bruce W. Watson, Kanshukan Rajaratnam, Maritha J. Kotze, Douglas B. Kell, Etheresia Pretorius

**Affiliations:** 1 School for Data Science and Computational Thinking, Stellenbosch University, Stellenbosch, South Africa; 2 David R. Cheriton School of Computer Science, University of Waterloo, Waterloo, Ontario, Canada; 3 Division of Chemical Pathology, Department of Pathology, National Health Laboratory Service, Tygerberg Hospital & Faculty of Medicine and Health Sciences, Stellenbosch University, Cape Town, South Africa; 4 Department of Biochemistry and Systems Biology, Faculty of Health and Life Sciences, Institute of Systems, Molecular and Integrative Biology, University of Liverpool, Liverpool, UK; 5 The Novo Nordisk Foundation Centre for Biosustainability, Technical University of Denmark, Lyngby, Denmark; 6 Department of Physiological Sciences, Faculty of Science, Stellenbosch University, Stellenbosch, South Africa

**Keywords:** Long COVID, data sharing, data science

## Abstract

‘Long COVID’ is the term used to describe the phenomenon in which patients who have survived a COVID-19 infection continue to experience prolonged SARS-CoV-2 symptoms. Millions of people across the globe are affected by Long COVID. Solving the Long COVID conundrum will require drawing upon the lessons of the COVID-19 pandemic, during which thousands of experts across diverse disciplines such as epidemiology, genomics, medicine, data science, and computer science collaborated, sharing data and pooling resources to attack the problem from multiple angles. Thus far, there has been no global consensus on the definition, diagnosis, and most effective treatment of Long COVID. In this work, we examine the possible applications of data sharing and data science in general with a view to, ultimately, understand Long COVID in greater detail and hasten relief for the millions of people experiencing it. We examine the literature and investigate the current state, challenges, and opportunities of data sharing in Long COVID research.

## Introduction

Post-acute sequelae of COVID-19 (PASC), otherwise known as ‘Long COVID’, is a health crisis resulting from the COVID-19 pandemic. In essence, Long COVID is the long-term reoccurrence of the symptoms and health challenges associated with a COVID-19 infection.^1–3^

**Figure 1:**
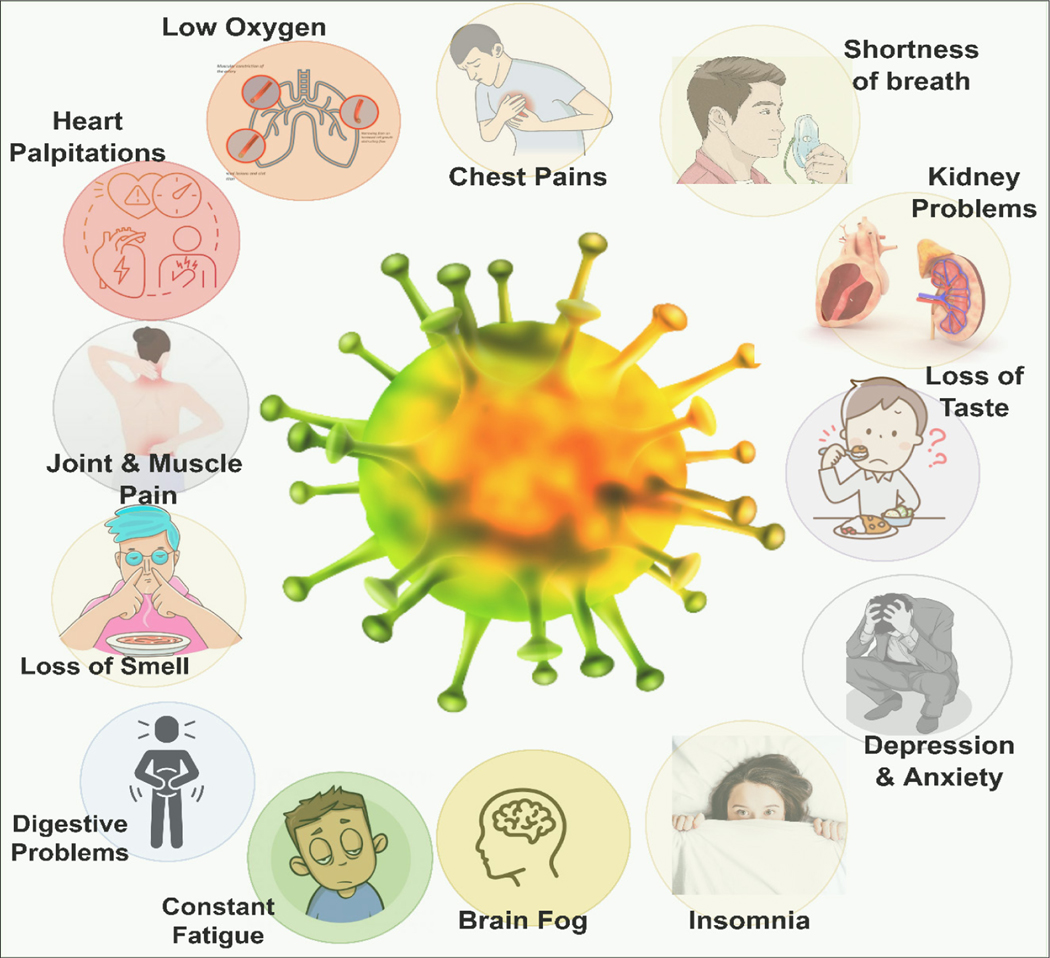
Illustration of the common Long COVID symptoms and complications reported in the literature.

Although the definition of Long COVID has initiated many complex conversations globally^4,5^, major Long COVID symptoms and complications agreed upon in the literature include: chest pain; heart palpitations; constant tiredness; muscular and joint pain; breathing difficulties (including low oxygen levels and shortness of breath); anosmia; difficulty concentrating; forgetfulness and brain fog; kidney problems; and digestive problems^3,6–8^ (Figure 1). COVID-19 survivors who still experience these persistent symptoms are called ‘Long haulers’.^9,10^

The severity and rate of occurrence of Long COVID symptoms in Long haulers differs depending on the patient’s health status prior to contracting COVID-19 and during treatment.^11^ Because of this, there remains considerable debate among medical professionals regarding how to make Long COVID diagnoses and what optimal treatment plans should look like.^12^ Disagreements and uncertainty often also result from the ways in which Long COVID data – post and prior to diagnosis (and treatment) – are collected, interpreted and reported.^13,14^ Data collection can be affected by the way that questions are phrased, the types of surveys used, and the potential biases of participants.^15^ Interpretation of the data can be affected by the way that they are presented, the types of analyses used, and the potential biases of the researchers. Reporting of the data can be affected by the way that data are summarised and the types of media outlets that are used, which can lead to miscommunication or confusion. As such, it is important to ensure that data collection, interpretation, and reporting are done in a transparent, unbiased manner in order to minimise disagreements and uncertainty. To this end, the processes involved in creating electronic health data and records must be more efficiently scrutinised and understood to avoid further muddying the waters.^11,14,16–18^ A single platform is required for data processing extending from sample/information collection to report generation.

The lack of a consistent definition for Long COVID has resulted in diverse data sets, with the further consequence of ambiguity in defining patients’ conditions and categorising based on patients’ conditions.^11^ Policies that define Long COVID can be improved in a variety of ways to better support Long COVID patients. First, there is a need to consider whether a new policy should be written, or rather be provided through an existing and appropriate form of management document. This would help healthcare providers to create standardised data collection and reporting systems that track Long COVID patient symptoms and health outcomes over time. These data could be aggregated and analysed to create a better understanding of the impact of Long COVID on patients, and to inform decisions about which treatments and interventions are most effective. The person responsible for keeping the data management plan or policy up to date must ensure that clear guidelines are provided for access and use in order to enforce adherence to the requirements. The lack of a standardised definition of Long COVID may also lead to unnecessary suffering on the individual level and exacerbates the existing strain on an already fragile global healthcare infrastructure and systems.

To establish effective and efficient management of Long COVID in patients, a standardised data capturing framework is therefore essential. A holistic data management framework would entail a wide-ranging collaboration across different specialities, drawing on research and expertise from a variety of sectors.^19^ In this paper, we examine the present challenges of applying data science and artificial intelligence (AI) to the problem, together with a consideration of other multidisciplinary approaches to solving the Long COVID conundrum.

## Data-driven frameworks in Long COVID management

Globally, healthcare organisations have accumulated several corpora of data from processes such as clinical workflows, drug trials, and patient medical records. These organisations are still, for the most part, utilising traditional approaches to recordkeeping and management. Traditional approaches to recordkeeping typically involve a paper-based system. This system includes the patient’s medical records, research data, and trial forms being entered into paper-based forms, notebooks, and logbooks. This system is often labour-intensive, but it is an effective method for collecting and organising data in a clinical trial. However, it can lead to inefficiencies in operations, such as poor patient admission and treatment and an overall sub-optimal management of and preparedness for epidemics and pandemics.^20,21^

A data-driven approach to healthcare management will improve on the efficiencies, agility, and robustness of healthcare institutions, enabling them to meet the intersecting challenges of increasingly complex patient needs and navigate the potential of ever-evolving medical technology in a dynamic global society. To achieve this goal, data science, AI, and information technology will play vital roles.^22–24^

**Figure 2:**
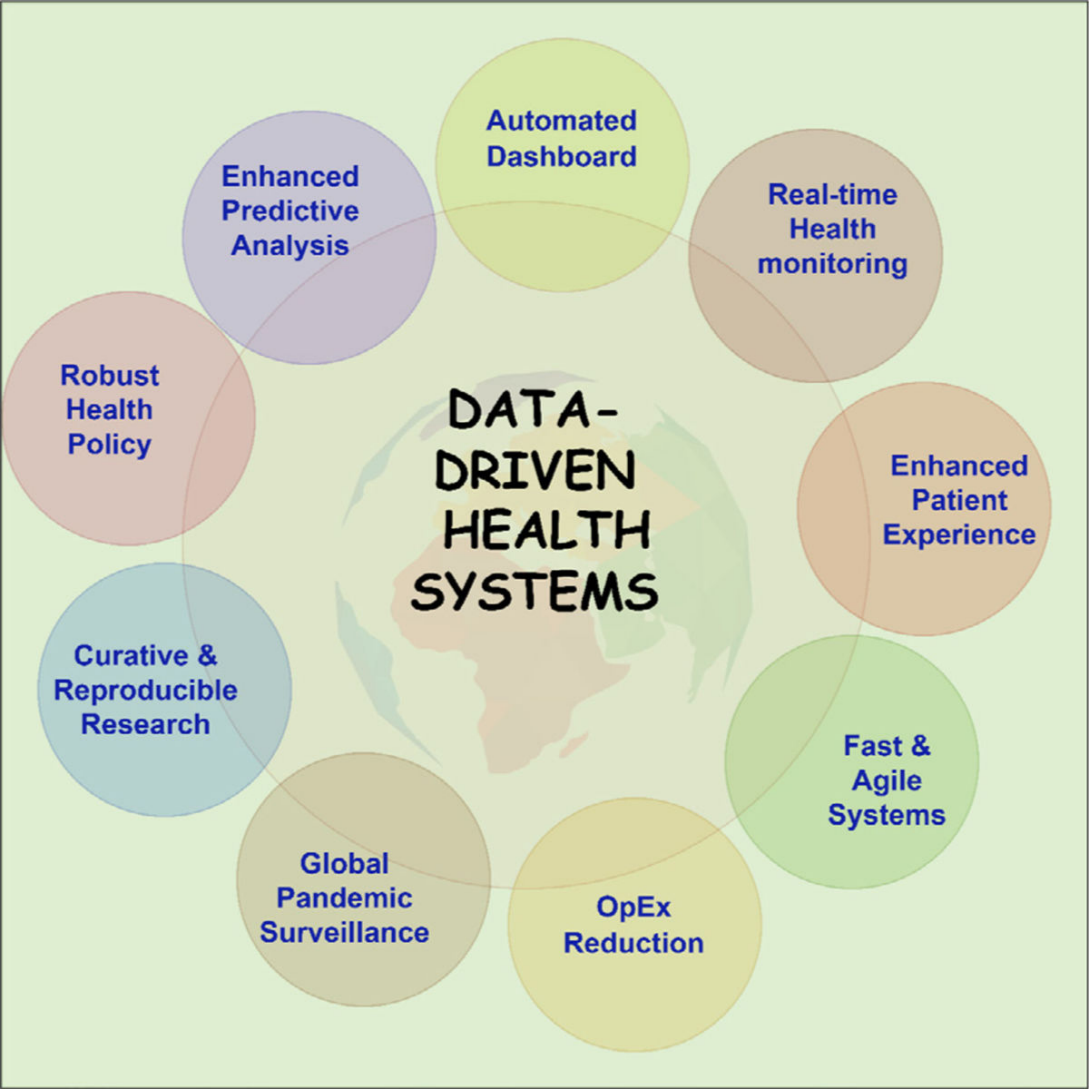
Benefits of adopting a data-driven framework for Long COVID management and healthcare systems in general.

Data-driven systems can also play a vital role in the management of Long COVID. Figure 2 illustrates some of the benefits of data-driven Long COVID management. However, there is a paucity of open big data sets for Long COVID management, which may be attributed to the novelty of the disease.^25^ Open big data sets are required by governments, healthcare institutions and policymakers across the world in designing capable healthcare systems to address the looming Long COVID crisis.^25^

The global move towards open science is largely seen as a positive development in the scientific community. Open science encourages the sharing of data, ideas, and methods, enabling researchers to collaborate more easily and efficiently. This promotes faster and more effective research and encourages the development of new approaches to research. Open science also allows for greater transparency and public engagement, as well as improved data accuracy and reproducibility. Ultimately, open science will help to ensure that scientific findings are as accurate and reliable as possible.^26,27^

In relation to Long COVID, the open science movement will be beneficial in helping researchers to collaborate and share data, which can be used to better understand the long-term effects of COVID-19. Open science can also provide a platform for patients to share their experiences and data, which can be used to inform further research. Furthermore, open data can be used to evaluate the effectiveness of treatments and develop new approaches to managing Long COVID. Ultimately, open science has the potential to advance our understanding of Long COVID and help to develop better strategies for prevention, diagnosis, and treatment.^13^

## Open big data sets for Long COVID

Data are a critical part of scientific research and the implementation of solutions proffered by researchers. Generally, data are also a major output in research endeavours, including clinical trials. Scientific data sets can be categorised as open sourced or closed source. Open-source data sets are available to everyone across the world without restriction. Open data sets support reproducible and collaborative research; enhance trust in research outcomes; and enforce best practices.^28^ Closed-source data sets are not made available to the public to protect intellectual property rights and privacy. Closed-source data sets include government-classified and privately owned data. Researchers who engage in restricting access to their data sets often do not share the base codes, methods, or techniques with the research community.

Data-driven systems and AI run on large data sets that are typically sourced from multiple sources and, hence, include open data sets but not exclusively so. Data science and AI played an important role in surveillance, treatment, and vaccination in the COVID-19 era, which was made possible due to data sharing among researchers and professionals globally.

However, the story is not the same for Long COVID, as there are only a few open-source data sets available on Long COVID surveys, clinical trials, and research. We carried out a text and meta search for Long COVID data sets online and in related published works, and found a total of 12 related data sets. Table 1 presents the outcome of our findings.

## Data sharing strategies

**Figure 3:**
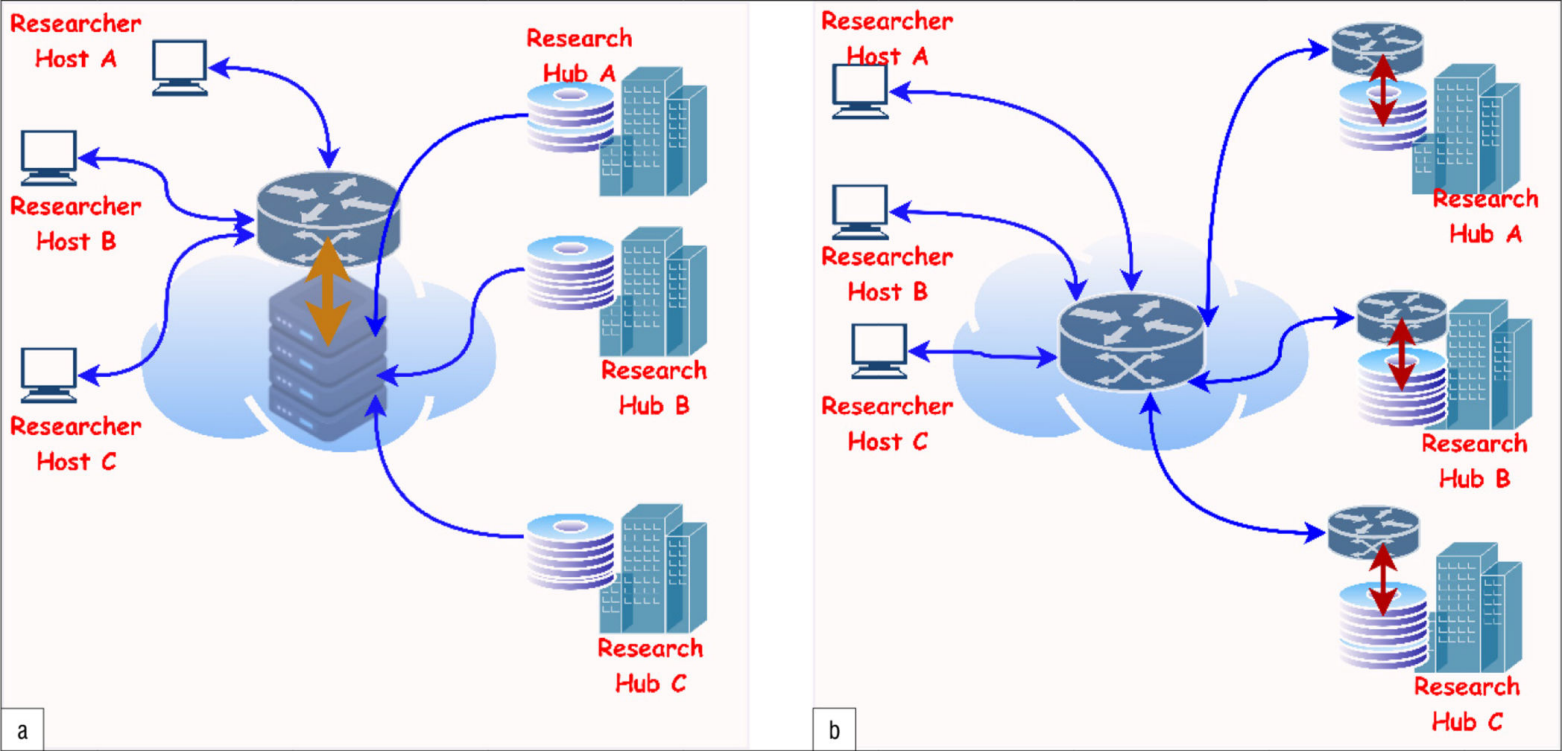
Illustration of the two main data-sharing strategies: (a) the centralised architecture and (b) the federated architecture.

To foster data sharing for Long COVID research, establishing effective data sharing strategies is important. In data sharing, for Long COVID and other health-related research, there are two broad storage strategies: (1) the centralised approach and (2) the federated approach. In the centralised repository approach, each respective research hub, community, or institution hosts and curates its data sets in one central data warehouse or storage facility, which connects to all other research hubs. Simply put, all research hubs store their data sets in the same data warehouse or repository. This architecture or approach is well suited for research purposes and research-generated data sets. In the federated approach, each respective research hub has its own data warehouse for data storage and other research hubs can only access the data sets via a web server. In the federated approach, restrictions can be enforced by the data sets’ owners due to data regulatory constraints and intellectual property rights. Each research hub is saddled with the responsibility of ensuring data privacy, security, and quality. The federated approach is well suited for electronic health data and records. Figure 3 illustrates the two approaches described above.

## Potential challenges in data sharing for Long COVID research

### Data availability and limitations

Owing to the novelty of Long COVID, there are few or, in some cases, no available data sets for researchers globally to compare notes. Moreover, the negligible quality of the available data sets may slow the process of finding appropriate solutions to Long COVID. The quality of a data set may, for instance, be undermined by the quality of available genomic sequences, unlabelled medical images, or low pixel resolution of medical images such as fluorescence microscopy and micrographs. Moreover, the population sizes of patients administered by a research community may also affect the generalisations and conclusions drawn from such studies.

The generalisation of AI-based medical systems is heavily reliant on the size and quality of the data used to train the system. With small data sets, it can be difficult to create an AI system that can generalise due to sample size issues, especially to new, unseen data. This is because small data sets can lead to a lack of diversity and a lack of statistical power, which can lead to overfitting and poor generalisation. Furthermore, small data sets can lack the necessary complexity to accurately capture the nuances of a medical problem. Therefore, when using an AI-based medical system, it is important to ensure that the data set used to train the system is large enough and of high enough quality to support accurate generalisation. Quality of data and data sets refers to a standardised definition of variables, and data sets that are difficult to harmonise. Moreover, creating AI models from data sets sourced from several research hubs or communities may be a daunting task, owing to different naming, file saving, and meta nomenclature, which could create serious problems when federating the data.

### Ethics, privacy, and security

**Figure 4:**
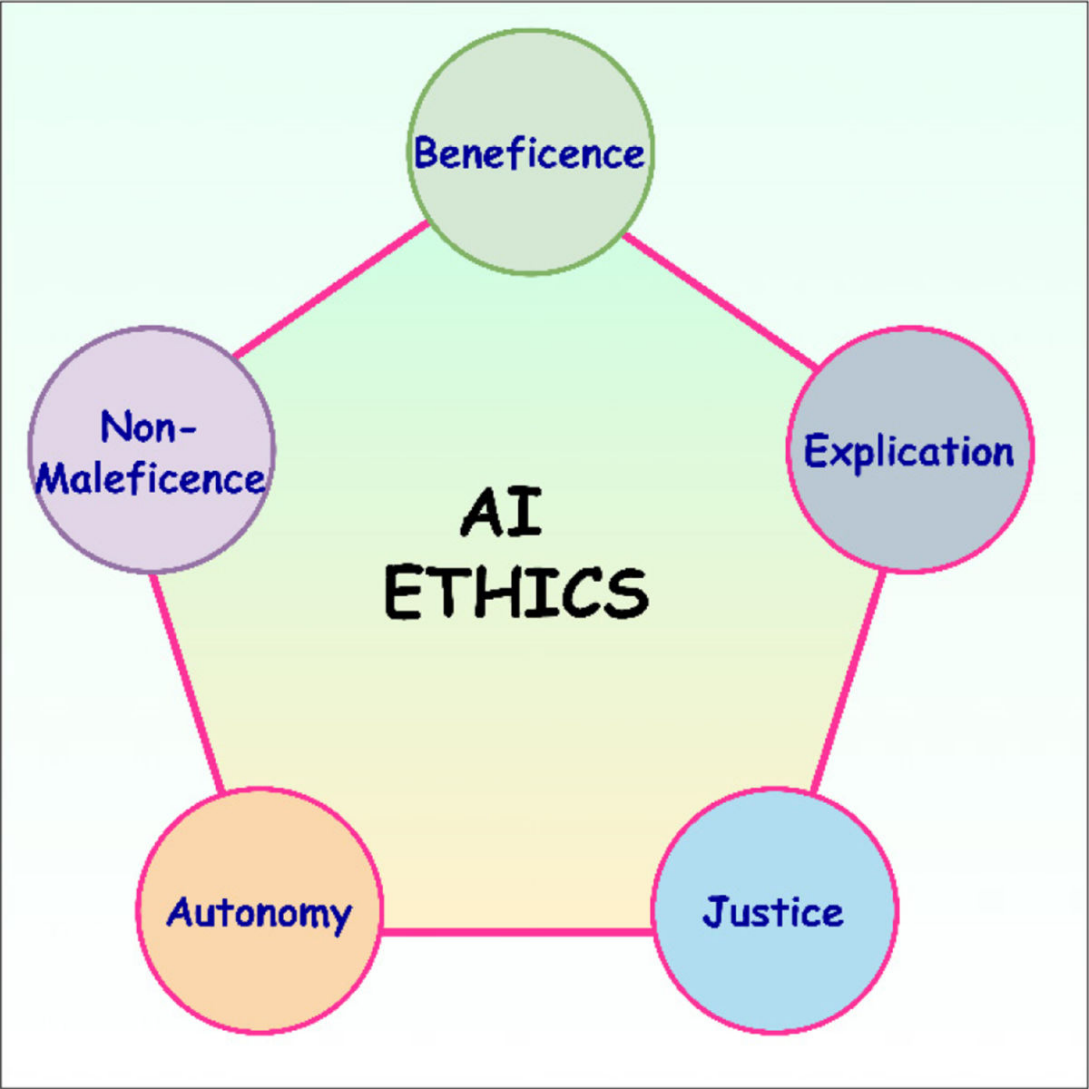
Pillars of artificial intelligence (AI) ethics.^43^

Ethics play a critical role in health sciences and medical professionals’ ability to provide safe and effective diagnoses and treatment for patients. Clinical trials should always adhere to best practices.^42^ COVID-19 and rising cases of Long COVID have initiated an intense discussion^12^ over how to find a compromise between the undeniable urgency of a globally accepted treatment, and the necessity of maintaining global best practices and ethics. In finding and achieving the desired balance, the quality of data sets from processes such as clinical trials in finding effective Long COVID treatment should not be compromised. Scientific rigour is essential for patient safety. Moreover, a data scientist must also adhere to AI ethics^43^, as illustrated in Figure4. In Figure 4, ‘explication’, also known as interpretability or explainability, is the transparency and the ability to understand how AI systems make decisions. For instance, an AI-powered medical diagnostic system that is opaque and not explainable could lead to mistrust among patients and healthcare providers. ‘Non-maleficence’ is closely related to the concept of safety in AI, in which AI-driven systems should not cause harm to humans or animals. For example, if an AI-powered medical diagnostic system misdiagnoses a patient, the patient could be harmed by receiving the wrong treatment. ‘Autonomy’ refers to the idea that individuals, communities, groups, and societies should have control over the use of AI systems that affect their lives. This principle is important to consider in AI development and deployment, as AI systems have the potential to make decisions that affect people’s lives in many ways, such as employment, health care, and criminal justice. Moreover, AI systems should be fair and not perpetuate or exacerbate existing inequalities; for example, an AI-powered criminal justice system that has been trained on biased data could lead to discrimination against certain groups of people. In order to ensure that the system is fair and does not make decisions that perpetuate existing inequalities, it is imperative that the data and data sets generated and studied do not possess or reproduce racial, gender, age, sexuality, religious, or disability-based biases. Likewise, the AI models developed from the data sharing effort must be devoid of biases.

### Sanctions and embargos on sharing information

Sanctions and embargos should not be placed on researchers and their respective home countries for sharing privacy-preserving Long COVID data sets, as this is both unreasonable and counterproductive. Such was infamously experienced by South African researchers as a consequence of their acting in the international community’s best interests by sharing their data on the SARS-CoV-2 Omicron variant.^44–46^ Travel restrictions put in place by the United Kingdom and other countries caused further damage to developing countries’ struggling economies while also worsening international relations. This incident generated discussions in research communities on the clear need to ensure that open science is not threatened. Long COVID researchers should be encouraged to look beyond narrow national interests and cultivate a global perspective in confronting Long COVID head on. Additionally, policymakers should consider long-term benefits of data sharing over narrow or irrational action which may result in short-term political benefits but hamper scientific discoveries and innovations. To illustrate this, globally, we now have two case studies to compare the consequences of sharing and not sharing data. In 2002, the Chinese government withheld SARS data and was severely criticised. However, travel bans were not enacted. This resulted in inadequate measures to prevent the virus spreading across borders.^47,48^ On the other hand, the South African government’s policy of open and transparent data sharing resulted in travel bans and restriction on freedom of movement.^47^ The latter had a negative impact on the economy and an adverse effect on import of much-needed medical products, resulting in further suffering. The negative reaction to South Africa’s sharing of data disincentivises countries from sharing data that may result in consequences for the global health system.^47^

Open science, virtual research collaborations, massive use of open access repositories, and agile research publication models should be encouraged, even in closed-border or travel-restricted situations.^49–52^ Open access publishing models should be encouraged to ensure that research results are accessible to all, regardless of geographical location.^51^

### Geopolitics of inclusivity and transparency

The geopolitics of global health have been a major determinant of whether people, nations, and continents have access to vaccines, patent waivers, and knowledge technology.^53–55^ As Long COVID patients are found across all countries, there is an urgent need for the discussions on diagnostic criteria, clinical trials, and treatment to be all-inclusive. To forestall the COVID-19 pandemic vaccine-hoarding phenomenon, developing countries should have their voices heard in the global conversation surrounding COVID-19 and be allowed to contribute their wealth of research and data. This will help to improve the accuracy and usefulness of models generated. Moreover, the developing world should not be treated as a monolith by wealthier nations. Surveys, clinical trials, and data-capturing processes should consider developing countries’ unique cultural, geographical, and political characteristics and how these might influence research at a micro and macro level.

### National and regional data regulatory frameworks

Ideally, national and regional regulatory frameworks should foster ethical data sharing and multinational collaboration. This is not usually the case, as data regulatory institutions and bodies enforce data protection laws which do not encourage data sharing. Concerning health-related issues, regulatory bodies are even stricter.^56^ There are technologies that allow for privacy-preserving sharing of data, which also protect to a large-extent the reverse engineering of such data sets to identify individuals or groups of individuals.^57^ Removing these barriers to privacy-preserving data-sharing would greatly encourage collaborative research for Long COVID.^58–60^

## Road map for the future: Health-related data sharing

The road map for health-related data sharing includes building health data science capacity, paradigm change in infrastructure, interoperability, and new governance and data ownership models.

### Health data science capacity building

To improve health-related data sharing among researchers and institutions health, the data science capacity of these researchers and institutions would need to be expanded.^61^ With health-related researchers and experts armed with the knowledge and importance of health data science, the culture of ethical data sharing and health data science would be embedded in the policies, operations, and processes such as clinical trials. To achieve this, the two other critical domains (i.e. computer science and mathematics/statistics) would need to be tailored to health-related professions in the health sciences curriculum globally. Moreover, all stakeholders, like health science educational standardisation institutions, would need to be engaged to see the importance of data science in uncovering insights into health-related diseases such as Long COVID and yet-to-happen pandemics. Additionally, health and medical practitioners should be encouraged (and mandated where/when necessary) to attend health data science trainings.^60,62–65^ Consequently, in the long term, data sharing and data science knowledge and skill sets would be imbibed in the medical and health sciences.

### Paradigm change in infrastructure

The global health industry sits on a vast amount of data such as electronic health data and records, genomic sequences, clinical trials, health surveys, and disease registries. To foster data sharing of health-related data sets, the mode and means of data set storage needs to be redesigned. Owing to the peculiarities of health-related data sets (such as privacy, security, and size), new technologies^66^ including blockchain, cloud storage, and quantum computing, should be embedded in the healthcare systems of the future. Blockchain and quantum computing can both help protect data and increase privacy and security. Blockchain technology is used to create an immutable, distributed ledger system that is secure and transparent (where transparency refers to the existence of the blockchain, while the actual data may be kept private). This system can help protect data from tampering and unauthorised access, while enabling users to control who has access to their data.^67–69^ Blockchain technology therefore enables privacy and security critical to health-related data sets. In addition, some aspects of quantum computing (specifically quantum information processing) can be used to secure data in two combinable ways. First, quantum key distribution (commonly known as QKD) uses quantum mechanics to create a secure and tamper-proof channel for data transmission, which is more secure than traditional encryption methods. Second, quantum-resilient cryptography (QRC, but also sometimes referred to as post-quantum cryptography, PQC) uses recently standardised algorithms – running on normal computers – that are practically impossible to crack, even with the help of the most powerful of computers.^67,70,71^ For instance, blockchain technology would enable privacy and security critical to health-related data sets.^72,73^ These technologies combined will play significantly critical roles in promoting data sharing and collaborative health-related research in future.

Soon, health-related research hubs and systems may outsource their data operations and management to technology-based corporations. This would allow health-related institutions and research hubs to leverage the computational and AI efficiencies of these specialised technology-savvy companies. To this end, the concept of health-data science/analytics as a service would dominate the discussions in the health industry.

### Interoperability

**Figure 5:**
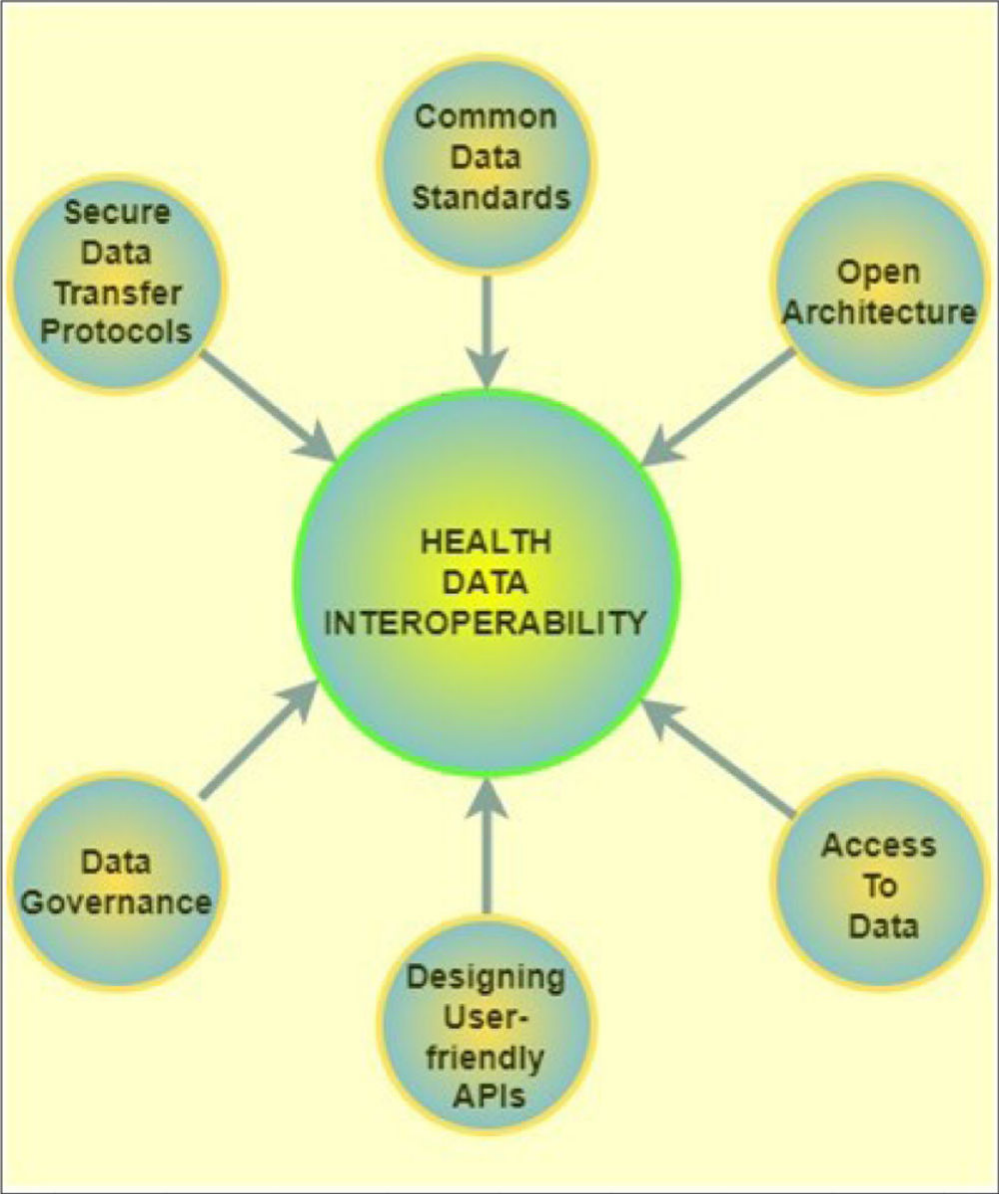
Key steps that contribute to the realisation of interoperability of health data.

Interoperability of data would play a critical role in sharing of health-related data. Interoperability, in this case, is the ability of stakeholders such as users, patients, their families, medical experts, and researchers to efficiently, securely, and timeously exchange health-related data with ease.^74^ Technologies such as blockchain enable interoperability that secures and allows for timeous exchange of health-related data. These technologies achieve interoperability through six main characteristics as depicted in Figure 5, which illustrates the factors that contribute to the realisation of health data interoperability. Interoperability is one of the main enablers of real-time data sharing of health information and data sets. Additionally, clinical trials and treatment of Long COVID will benefit from the transparency fostered by the interoperability of data sharing. There is no doubt that interoperability will promote a nationwide, internation, and global-wide data-sharing culture.^75^

### New governance and data ownership models

The discussion around data ownership determines the ease with which, how, where, and what type of data are captured, stored, and shared. Currently, health institutions and research hubs believe that their own patients’ data are in their custody.^76^ On the contrary, patients are increasingly aware of their data rights and, consequently, demand consent before their data are used. New governance and owner models would greatly forestall legal bottlenecks to efficient data sharing that may arise from data ownership. Data governance and ownership models (such as data sharing pools, data cooperatives, public data trusts, and personal data sovereignty) as a future road map for health data sharing have been discussed in the literature.^77–79^ Fulfilling data regulations such as POPIA and GDPR, although onerous, require consent from patients and should be integrated in both existing and future systems.^80^

### Data sharing templates and agreements

Sharing medical and health-related data raises concerns about the ethical use of data sets. To forestall future legal issues and ensure the ethical use of data sets, a data sharing template and agreement should be used by the data custodians. Data sharing templates and agreements may help assuage the fears of data custodians who are not ready or willing to make their data sets open to the public by rethinking ‘on reasonable request’. The data sharing template and agreements will provide a guide from scientific discovery to clinical application of our current knowledge about the pathogenesis of Long COVID. A readiness checklist including the requirement of a data sharing agreement for implementation of genomic medicine programmes involving return of research results at the intersection of research and service delivery is given by Jongeneel et al.^81^ Although data sharing templates and agreements are not new in medical research, Long COVID research is relatively in its early stages. Data sharing templates and agreements designed for COVID, if invested in, would significantly help to foster data sharing among Long COVID researchers.

### Clinical policymakers as gatekeepers

Data sharing should create value that benefits adopters^82^, i.e. generators of the data. Clear benefits create incentives to move from few adopters to mainstream practices. We posit that clinical policymakers are the gatekeepers of information flow from clinical research to best practice policy in a patient setting. Given the incentive for clinical researchers to impact on patient treatment practices, clinical policymakers are in a position to create incentives for data sharing. Clinical policymakers may provide incentives within the requirements for successful research funding and grants to support clinical research, through recognition, and through the promotion of their research at the institutional or national level, as well as through academic recognition in the form of awards and publications. Additionally, clinical researchers may be incentivised by professional satisfaction when they see their research directly impacting patient care and clinical practice. Moreover, there are inherent advantages of data sharing to both clinical researchers and policymakers such as enhancing transparency and public trust. Clinical policymakers have the opportunity to increase diffusion of data-sharing practices among data-generating researchers by ensuring best practices with respect to data sharing are followed during the clinical research that results in patient treatment policies. These best practices can be ensured by: establishing clear policies and procedures for data sharing that outline the expectations; providing training and education for clinical researchers on data sharing best practices; monitoring and auditing (including periodic reviews of) data sharing activities; encouraging collaboration among clinical researchers; and utilising data sharing platforms and services that provide secure and efficient ways to store and share data. This is analogous to mortgage lenders being the gatekeepers to encourage uptake of energy-efficient homes.^83^

## Conclusion

Despite millions of people across the world having been diagnosed with Long COVID, and the detrimental impact on the health and wealth of individuals and economies, there have been few global concerted efforts to encourage data sharing and data science to uncover insights into this disease. In this paper, we examined the benefits of data-driven frameworks, in particular open big data sets, for Long COVID. Moreover, a review of the research data set and the current state of data sharing was carried out on Long COVID research in Africa and the world in general. To encourage data sharing and collaborative Long COVID research, we examined potential challenges and also discussed the road map for the future of health data sharing.

## Figures and Tables

**Table 1:** Related Long COVID data sets in the literature

Study	Country of study/participants	Number of participants	Mode of data sourcing	Duration of study	Data availability
Patient-led Research Collaborative^29^	56 Countries	3762	Online survey	6 Sep 2020 – 25 Nov 2020	On request
SA Long COVID^6,30^	South Africa	845	Online survey		–
Long COVID Support Group^31^	United Kingdom	114	Physical interview and focus group	May 2020 – Sep 2020	–
Schools Infection Survey Long COVID^32^	England	3779 Primary 2961 Secondary	Questionnaire	15 Mar 2022 – 1 Apr 2022	Available
Hiroshima Prefecture Survey^33^	Hiroshima, Japan	140	Self-administered questionnaire	25 Aug 2020 – 15 Mar 2021	On reasonable request
ZOE COVID-19 Tracker^34^	United Kingdom, USA, Sweden	4182	Phone app (self)	24 Mar 2020 – 2 Sep 2020	–
Symptom Burden Question for Long COVID (SBQ-LC)^35^	United Kingdom	274	Remote data collection and social media channels	14 Apr 2021 – 1 Aug 2021	–
DATCOV Post COVID Condition^36^	South Africa	1873	–	1 Dec 2020 – 23 Aug 2021	–
Long COVID Dataverse^37^	United Kingdom, Lesotho, Angola, Israel, USA	1131	–	Mar 2022	Available
Self-Reported Long COVID after Omicron^38^	United Kingdom	–	–	18 Jul 2022 – 6 May 2022	Available
Prevalence of Ongoing COVID19 Symptoms^39^	United Kingdom	–	–	1 Apr 2021 – 7 Jul 2022	Available
Kenya, Malawi, Long COVID effect survey^40^	Kenya Malawi	806 Kenya 885 Malawi		6 Sep 2021 – 2 Oct 2021	Available
American Academy of Physical Medicine (AAPM&R)^41^	USA	–	–	From July 2021	–

## Significance:

Although millions of people across the globe have been diagnosed with Long COVID, there still exist many research gaps in our understanding of the condition and its underlying causes. This work aims to elevate the discussion surrounding data sharing and data science in the research community and to engage data sharing as an enabler to fast-track the process of finding effective treatment for Long COVID.
